# Inter-individual variability in performance benefits from repeated sprint training in hypoxia and associated training parameters

**DOI:** 10.3389/fspor.2025.1524437

**Published:** 2025-04-15

**Authors:** Naoya Takei, Ryuji Muraki, Olivier Girard, Hideo Hatta

**Affiliations:** ^1^ Research Institute of Physical Fitness, Japan Women’s College of Physical Education, Tokyo, Japan; ^2^ Department of Sports Sciences, The University of Tokyo, Tokyo, Japan; ^3^ Department of Sports Science, Surugadai University, Saitama, Japan; ^4^ School of Human Sciences (Exercise and Sport Science), University of Western Australia, Perth, WA, Australia

**Keywords:** inter-individual variability, simulated altitude, non-responder, oxygen saturation, repeated sprint training in hypoxia

## Abstract

This study examined whether inter-individual variability exists in repeated sprint training in hypoxia (RSH) and how peripheral oxygen saturation (SpO_2_) affects physiological demands and mechanical output, and subsequent training outcomes. Sixteen highly-trained sprint runners completed six sessions of RSH consisting of two sets of 5 × 10-s all-out sprints (fraction of inspired oxygen: 0.15), with pre- and post-tests involving 10 × 10-s all-out sprints in normoxia. Average SpO_2_, training impulse (TRIMP), and relative total work (relative TW; standardized by pre-test TW) during training sessions were calculated. After the intervention, MPO increased by +3.8% (*P* = 0.001) and sprint decrement score by +6.0% (*P* = 0.047). However, inter-individual variability in performance improvement observed and nearly 20% of participants did not obtain performance benefit. Average SpO_2_ during training sessions correlated significantly with relative TW (*r* = 0.435, *P* = 0.008), indicating that participants with higher SpO_2_ performed more work during training. Relative TW was strongly correlated with performance improvement (*r* = 0.833, *P* < 0.001), suggesting that those who produced more work during training experienced greater performance gains. TRIMP showed no significant correlation with SpO_2_ or performance improvement. In summary, greater peripheral deoxygenation leads to lower mechanical work and consequently smaller performance improvement following RSH. The variability in peripheral deoxygenation and relative TW among highly-trained sprint runners may contribute to the heterogeneous training effects observed.

## 1 Introduction

Altitude/hypoxic training is widely used by elite athletes (1, 2). Repeated sprint ability (RSA), the ability to repeatedly perform all-out or near maximal efforts with incomplete recoveries, is crucial for team and racket sports (3). Repeated all-out sprints (<10 s) with short incomplete recoveries (<60 s) in hypoxia (repeated sprint training in hypoxia, RSH) effectively improve RSA (4–6). Consequently, RSH is widely used by athletes engaged in these activities (4, 5). Previous studies have shown that RSH elicits significant physiological adaptations and improves RSA compared with equivalent normoxic training (4, 6). A meta-analysis demonstrated that RSH significantly increases peak (SMD = 0.31) and mean (SMD = 0.46) power outputs, confirming that this intervention boost RSA (4). Although hypoxic training, particularly RSH, induces positive performance adaptations, inter-individual variability in training adaptations remains unclear. Recent suggestions indicate that even with the same fraction of inspired oxygen (FiO_2_), physiological responses may exhibit inter-individual variability, potentially influencing training outcomes (7). Therefore, it is recommended to monitor physiological parameters, such as arterial oxygen saturation (SpO_2_) for personalized hypoxic training prescriptions (7). However, it remains unclear whether there is inter-individual variation in response to RSH.

Inter-individual variation in arterial oxygen saturation (SpO_2_; internal hypoxic response) exists when identical hypoxic exposure (external hypoxic stimulus) is applied (8, 9). This variation in SpO_2_ has been linked to acute performance responses (3,000-m time trial) under moderate hypoxia (∼2,100 m above sea level), where individuals with larger arterial deoxygenation exhibited greater performance declines (10). Likewise, our previous study (11) found that inter-individual variability in SpO₂ under identical hypoxic conditions (FiO_2_: 0.150) influences RSA (10 × 10-s all-out sprints with 30-s recovery), with greater deoxygenation impairing performance. However, it remains unclear whether SpO₂ fluctuations influence training effects. Although not specifically examining SpO₂ variability under identical hypoxic conditions, Gutknecht et al. (12) reported that SpO₂ fluctuations during RSH training sessions were significantly correlated with performance improvements after two weeks of RSH at different hypoxia levels (FiO₂: 0.141, 0.162, or 0.175), suggesting that performance improvements were diminished as SpO₂ levels decreased. Taken together, these studies highlight substantial inter-individual variation in internal responses to the same external hypoxic stimulus, which may influence absolute training intensity and, consequently, training outcomes.

Training effects are influenced by both physiological load and mechanical output (13). Training in hypoxia can cause pronounced arterial (i.e., hypoxemia) and tissue deoxygenation (e.g., deoxygenation in working muscles), activating signaling pathways such as hypoxia-inducible factor-1 (HIF-1) (14), which promotes physiological adaptations, including increased angiogenesis and enhanced energy metabolism, ultimately improving exercise performance (6, 15, 16). However, excessive arterial deoxygenation (hypoxemia) can impair exercise performance by reducing absolute training intensity (10, 11, 17). Studies suggest that lower absolute exercise intensity leads to decreased mechanical stimulus, potentially limiting training effects (18–20). Therefore, balancing physiological demands and mechanical output is crucial for optimal training outcomes. To date, there is limited evidence on the influence of inter-individual variability in response to hypoxia on physiological demands, mechanical output, and subsequent RSH training outcomes.

Individual responses to training interventions are believed to vary considerably. For personalized training prescriptions, it is necessary to identify “responders” and “non-responders” and understand the factors behind these differences. However, much of the observed inter-individual variability may reflect “apparent differences” due to measurement errors (21–23). To accurately assess inter-individual variability, randomized controlled trials with comparison groups are required to adjust for random errors (21, 22). In studies involving athletes, however, it is often difficult to include a control group receiving no intervention. This single-arm study targeted highly trained athletes, but we conducted a test-retest reliability assessment to estimate random measurement noise and evaluate inter-individual variability (21).

This study aimed to test the hypothesis that inter-individual variability in internal hypoxic response (SpO_2_) influences exercise performance (mechanical output), leading to heterogeneous training outcomes. Specifically, this study aimed to (1) determine whether inter-individual variability exists in training effects from a RSH intervention, and (2) identify potential factors (SpO_2_, heart rate, mechanical output) associated with this variability*.* We also hypothesized that participants with greater SpO_2_ decreases during RSH would experience larger performance decrements (reduced mechanical output), potentially resulting in minimal or no training benefits, and *vice versa*.

## 2 Methods

### 2.1 Participants

The sample size was determined using power analysis software (G*Power 3.1.9.7, Heinrich-Heine-Universität Düsseldorf, Germany; 1-*β* = 0.80, *α* = 0.05) based on the mean effect size (*r* = 0.61) for the correlation between SpO_2_ and exercise performance (sprint decrement score) during hypoxic repeated sprint exercise (11). The power analysis calculated a total sample size of 13 participants, and 16 were recruited to account for potential dropouts. Sixteen male, highly-trained sprint runners (Age: 20.2 ± 1.7 year; Weight: 66.9 ± 1.7 kg; Height: 1.74 ± 0.07 m), categorized as Tier 3 by established criteria (24), volunteered after providing written informed consent. Participant characteristics are displayed in Supplementary Table S1. All participants were born and lived near sea level and had not been exposed to hypoxic environments in the previous three months. The study adhered to the Declaration of Helsinki and was approved by the Research Ethics Committee of the University of Tokyo (No. 891).

### 2.2 Design and procedures

This observational study investigated inter-individual variability in training effects as well as the physiological and mechanical training load of RSH. The intervention involved two weeks of RSH (two sets of 5 × 10-s all-out sprints with 30-s recovery; three times per week) in hypoxia (FiO_2_ = 0.15). Performance tests (10 × 10-s all-out sprints with 30-s recovery) were performed before and after the intervention (pre- and post-test) in normoxia (FiO_2_ = 0.21). Although six sessions of RSH training is a common protocol, the training volume in this study was relatively small for sprint runners, with only 2 sets compared to 3–4 sets used in previous studies involving other athletic cohorts [e.g., team sports, cycling, cross-country skiing athletes; (4)]. All participants belonged to the same track and field club and followed a similar training regimen during the experimental period. To compare inter-individual differences in training effects, the magnitude of pre-post mean power improvements (%MPO improvement) was calculated. Furthermore, performance and physiological parameters for each training session were analyzed and compared with training outcomes to identify factors influencing inter-individual variability in RSH effects.

Training and testing used an electrically braked cycle ergometer (PowerMax VIII, Konami, Japan), with workloads set at 5.0% of each participant's body weight. Handle and seat positions were replicated for all sessions. Before each session, participants performed a self-selected warm-up (e.g., walking, jogging, dynamic stretching), followed by 3 × 10 s cycle sprints (inter-sprint recovery = 50 s) at increasing effort levels (60, 80% and 100% of voluntary maximal effort) in hypoxia (FiO_2_: 0.15). The training intervention consisted of six sessions of repeated sprint training (two sets of 5 × 10-s all-out cycle sprints; recovery: inter-sprint = 30 s, inter-set = 5 min) in hypoxia (FiO_2_:0.15) over two weeks (three times per week). Given the participants' lack of familiarity with all-out sprinting on a treadmill and to eliminate the risk of falls or injuries and reduce the total amount of ground contact by running, we opted for all-out sprint cycling. All participants regularly incorporated all-out sprint cycling as part of their training routine. Throughout the intervention, participants maintained their regular track and field training regimen (two to three hours per session, five sessions per week). Performance tests were conducted before and after the training intervention, involving 10 × 10-s all-out sprints with 30-s recovery, in normoxia (FiO_2_ = 0.21). All tests were conducted at ∼93 m above sea level, with test and training sessions spaced two to three days apart. Participants were asked to avoid ergogenic substances (e.g., energy drinks, supplements) for 24 h before testing and refrain from heavy physical activity for 48 h prior. All tests were conducted at the same time of day (±1 h) for each participant to minimize circadian influences.

### 2.3 Measures

The hypoxic chamber (FCC-5000S, Fuji Medical Science, Japan) maintained a regulated normobaric hypoxic environment (15% O_2_ and <1% CO_2_) through a nitrogen dilution technique. Testing utilized an electrically braked cycle ergometer (PowerMax VIII, Konami, Japan) to measure mean power output (MPO) for each sprint. The sprint decrement score (S_dec_) was calculated as described previously (3). Heart rate (Polar H10, Polar, Finland) was measured immediately before the first sprint and ∼10 s after each sprint. For assessing physiological training loads, averaged post-sprint SpO_2_ (SAT-2200, Nihonkohden, Japan) were recorded as an internal hypoxic stimulus, and training impulse (TRIMP) was calculated using heart rate data via the banister's method (25). To standardize performance across individuals, standardized total work (relative TW) was calculated by dividing the total workload during training in hypoxia by the total workload (TW) measured during the pre-test in normoxia. Blood lactate concentrations (BLa) were measured from fingertip blood samples using a portable lactate analyzer (Lactate Pro 2, Arkrey, Japan), collected 5 min after the last sprint in both the pre- and post-tests.

This study examined inter-individual variability in the training effects of RSH interventions. To accurately assess inter-individual variability, it is necessary to account for intra-individual variability and measurement errors from the observed changes. Typically, a control group is required to adjust for these factors; however, due to ethical and logistical considerations, this was a single-arm study targeting highly trained athletes (21, 22). In line with previous recommendations, we conducted test-retest measurements to assess the reliability of pre- and post-intervention measurements, quantifying measurement error and intra-individual variability (21, 26). The test-retest was performed with 12 participants who had similar profiles to those in the main experiment (highly trained sprint runners) and experienced with cycling sprints. The same measurement devices, environmental conditions, and procedures were used, with a two-week interval. Statistical methods for assessing inter-individual variability are detailed in previous studies (21, 22).

### 2.4 Analysis

Statistical analysis was conducted in GraphPad Prism (v10.2.2; GraphPad Software, USA). Data normality was assessed with the Shapiro–Wilk test, and if violated, the Wilcoxon signed-rank test was applied. Paired *t*-tests (pre vs. post) and two-way repeated measures ANOVA [repetition (sprint 1, 2, 3, …, and 10) × time (pre and post)] were used to compare dependent variables, followed by Tukey's multiple comparisons. Mauchly's sphericity test was employed to assess the assumptions of variance for all ANOVA results, with a Greenhouse–Geisser correction applied when necessary. Pearson's correlation coefficients were computed to investigate relationships between variables. Effect sizes were determined using Cohen's *d* (0.20–0.49 = *small* effect; 0.50–0.79 = *moderate* effect; and ≥0.80 = *large* effect) or eta squared (*η*^2^; 0.010–0.059 = *small* effect; 0.060–0.139 = *moderate* effect; and ≥0.140 = *large* effect). Data are presented as mean ± standard deviation (SD), with statistical significance set at *P* < 0.05.

To assess inter-individual variability, SD of individual responses (SD_IR_) was calculated using the following equation (21, 22):

$$SD_{IR}=\sqrt{SD_{I}^{2}-SD_{CTRL}^{2}}$$

where SD_I_ and SD_CTRL_ represent the SD of change scores from training intervention result (SD_I_) and test-retest result (SD_CTRL_), respectively. A positive SD_IR_ indicates inter-individual differences in training adaptations, while an indeterminate or negative SD_IR_ suggests no meaningful differences. The proportion of responders and non-responders is determined using a normal distribution model, centered on the mean change score from the intervention, with a standard deviation of SD_IR_. This proportion is calculated as the area of normal distribution that exceeds the smallest worthwhile change, which is defined as 20% of the baseline standard deviation of the training intervention (22).

## 3 Results

**Figure 1:**
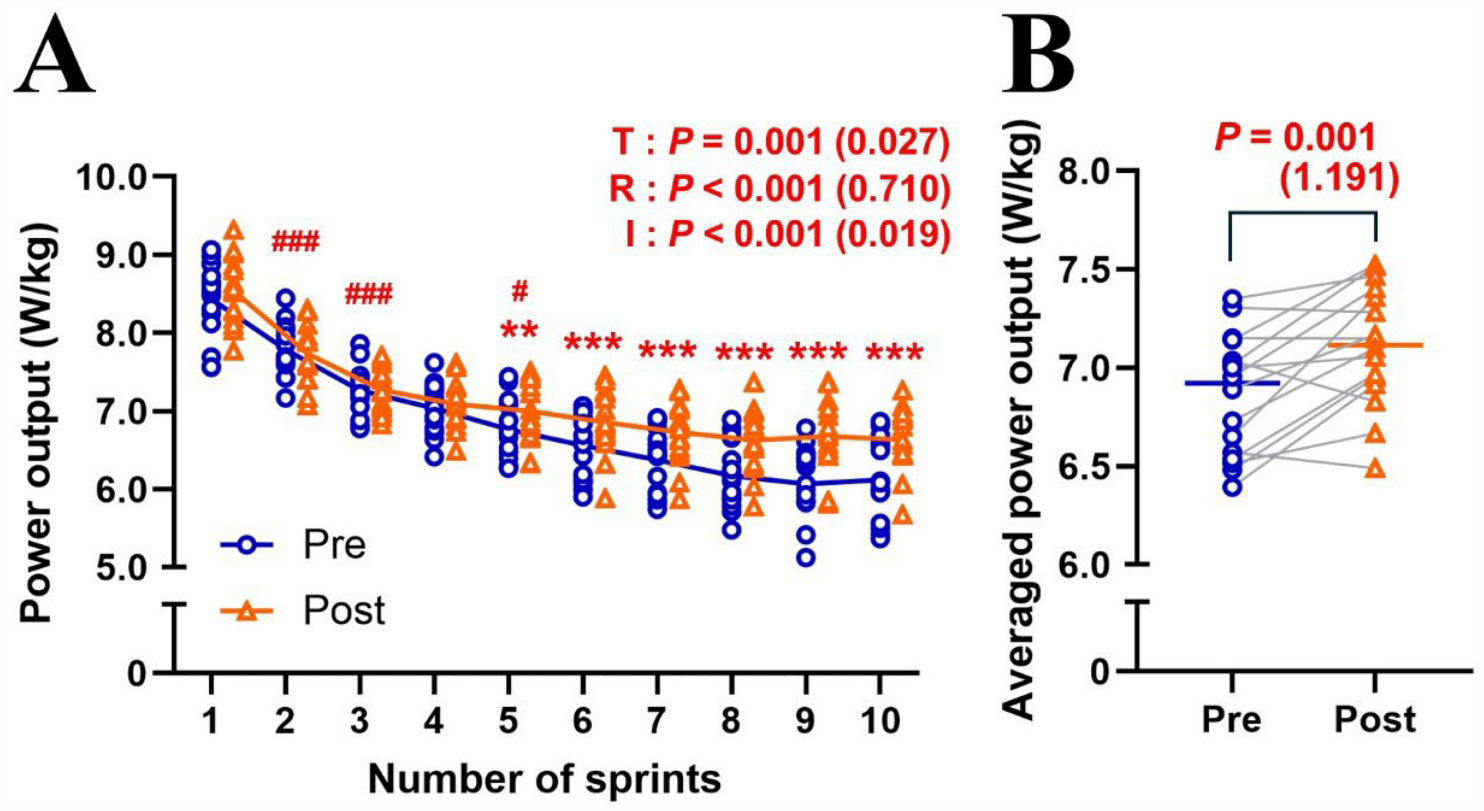
Changes in mean power output across sprint repetitions **(A)** and averaged mean power output of ten sprints **(B)** for pre- and post-tests. T, training; R, repetition; I, interaction. Blue circle markers and line indicate the pre-test, while orange triangles and line indicate the post-test values. Markers represent individual values (*n* = 16), and lines represent mean values. Gray connecting line indicate individual changes between pre- and post-test. Paired *t*-test (pre × post) and two-way repeated measures ANOVA [repetition (sprint 1, 2, 3, …, and 10) × time (pre and post)] were applied. ###*P* < 0.001, #*P* < 0.05, significantly different from the previous sprint (i.e., sprint 1 vs. 2; sprint 2 vs. 3; sprint 4 vs. 5). *** *P* < 0.001, ** *P* < 0.01, significantly different between pre- and post-tests. *P* value and effect size are expressed as *P* value (effect size).

**Figure 2:**
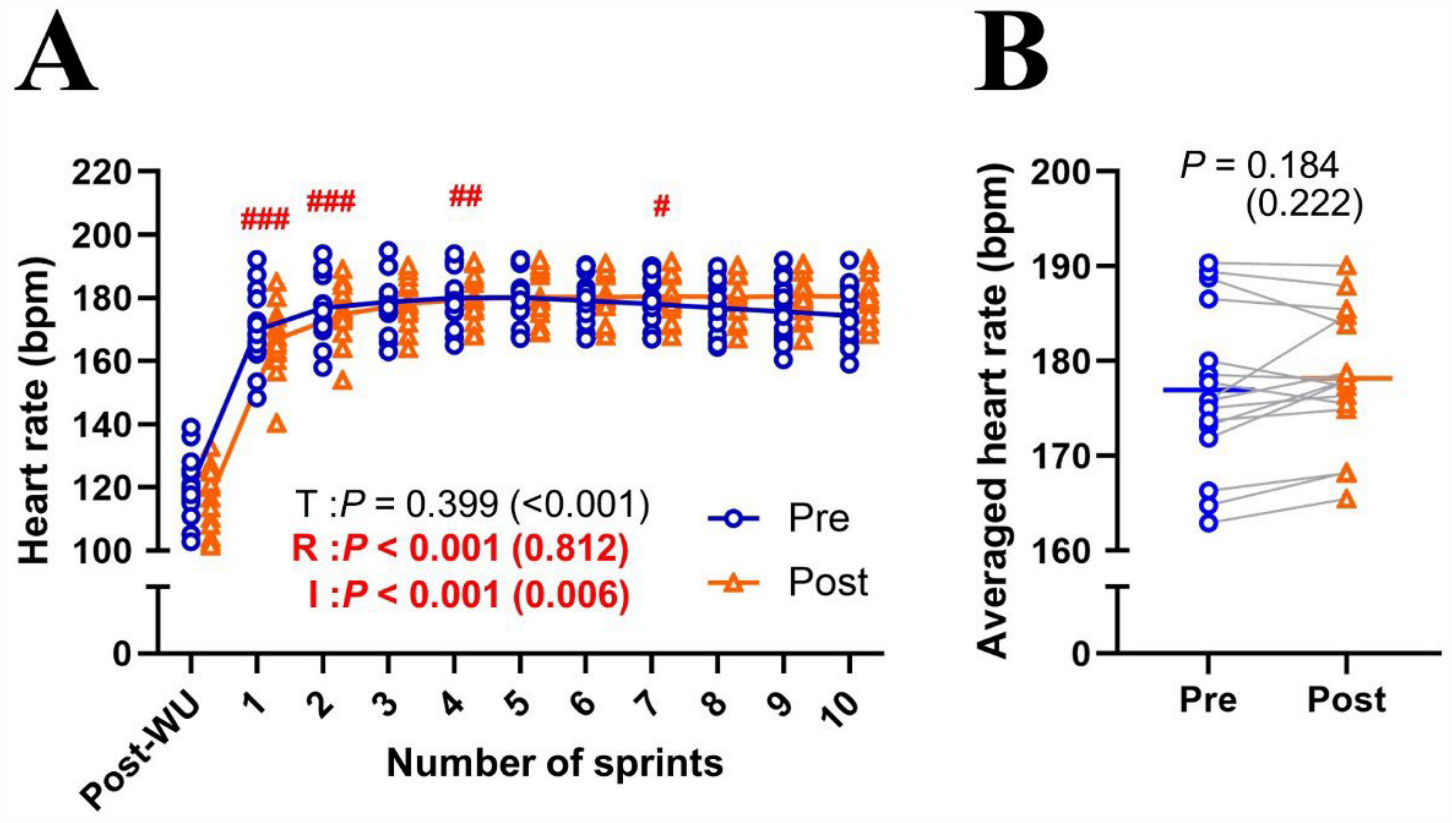
Changes in heart rate across sprint repetitions **(A)** and the heart rate of ten sprints **(B)** for pre- and post-tests. T, training; R, repetition; I, interaction; post-WU, post-warm up. Blue circle markers and line indicate the pre-test, while orange triangles and line indicate the post-test values. Markers represent individual values (*n* = 16), and lines represent mean values. Gray connecting line indicate individual changes between pre- and post-test. Paired *t-*test (pre × post) and two-way repeated measures ANOVA [repetition (post-WU, sprint 1, 2, 3, …, and 10) × time (pre and post)] were applied. ###*P* < 0.001, ## *P* < 0.01 #*P* < 0.05, significantly different from the previous sprint (i.e., post-WU vs. sprint 1; sprint 1 vs. 2; sprint 3 vs. 4). *P* value and effect size are expressed as *P* value (effect size).

**Figure 3:**
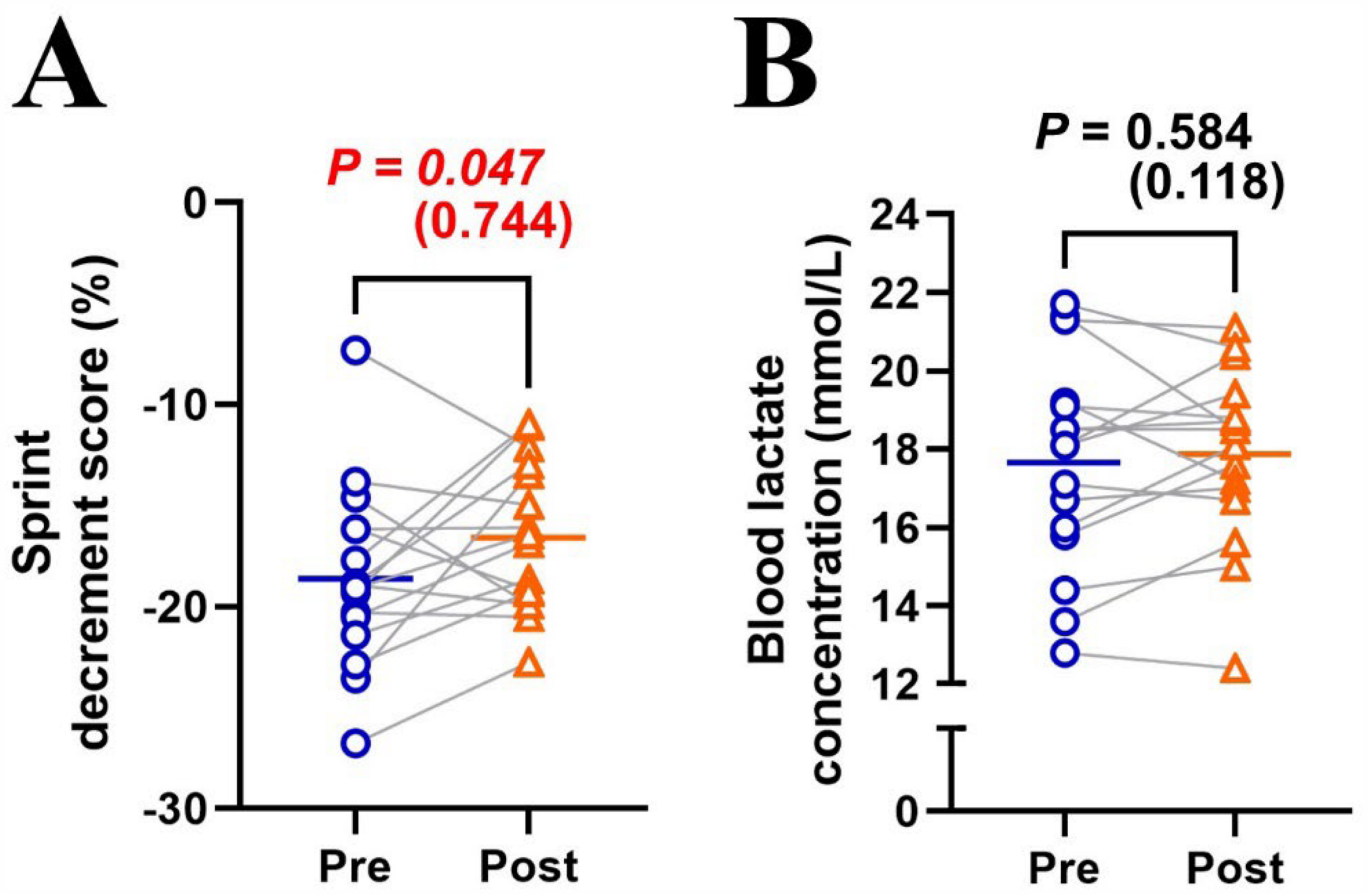
Changes in sprint decrement score **(A)** and blood lactate concentration **(B)** between pre- and post-tests. Blue circle markers and line indicate the pre-test, while orange triangles and line indicate the post-test. Markers represent individual values (*n* = 16), and lines represent mean values. Gray connecting lines indicate individual changes between pre- and post-tests. Paired *t*-test (pre × post) was applied.

MPO significantly decreased (Δsprint 1–10: −24.7 ± 6.4%; *P* < 0.001; *η*^2^ = 0.710) across repetitions and improved (+3.8 ± 3.9%; *P* = 0.001; *η*^2^ = 0.027) after the training intervention (Figure 1A). *post hoc* tests revealed that MPO significantly increased (all *P* < 0.01) in the later sprints (sprints 5–10) in post-test compared to pre-test (Figure 1A). Averaged MPO was significantly greater (*P* = 0.001; *d* = 1.191) in post-test than in pre-test (7.11 ± 0.30 vs. 6.85 ± 0.31 W/kg; Figure 1B). Heart rate significantly increased across repetitions (Δpost-WU-sprint10: +50.2 ± 13.0%; *P* < 0.001; *η*^2^ = 0.812), irrespectively of training (*P* = 0.399; *η*^2^ < 0.001; Figure 2A). Averaged heart rate did not differ (*P* = 0.184; *d* = 0.222) between pre- and post-tests (177.0 ± 8.5 vs. 178.2 ± 7.1 bpm; Figure 2B). S_dec_ was significantly improved (−18.6 ± 4.5 vs. −16.3 ± 4.2%; *P* = 0.047; *d* = 0.744) after the intervention (Figure 3A), while BLa (*P* = 0.584; *d* = 0.118) did not differ between pre- and post-tests (17.6 ± 2.7 vs. 17.9 ± 2.2 mmol/L; Figure 3B).

To estimate random noise, the averaged MPO for the test and retest were identified as 7.12 ± 0.69 W/kg and 7.12 ± 0.65 W/kg, respectively. The SD of the change score for the test-retest (i.e., SD_CTRL_) was 0.139 W/kg, while the SD of the change score for the intervention (i.e., SD_I_) was 0.261 W/kg. The calculated SD_IR_ was 0.221 W/kg, indicating inter-individual variation. The proportion of responders was 81.3%, suggesting that nearly 20% of participants did not experience a performance benefit from the intervention.

**Figure 4:**
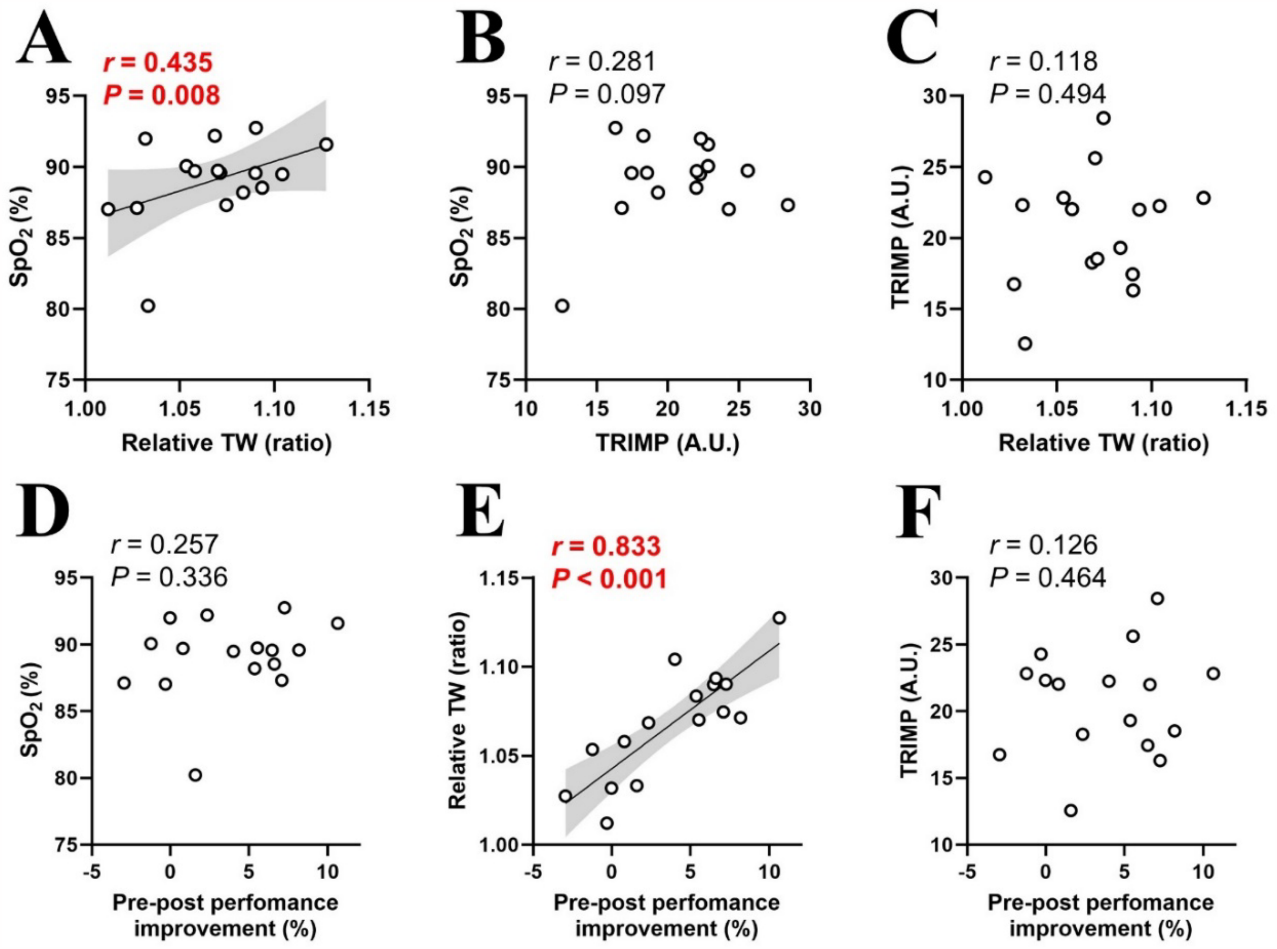
Relationships between physiological and mechanical training loads **(A–C)**, and pre-post performance improvements **(D–F)**. SpO_2_, arterial oxygen saturation in hypoxia; TW, total work; TRIMP, training impulse. Circle markers indicate individual measured values (*n* = 16), and solid lines indicate approximate straight lines. Pearson's correlation coefficients were computed to investigate relationships between variables. The gray area represents the 95% confidence interval.

A significant correlation was noted between SpO_2_ in hypoxic condition and relative TW (*r* = 0.435, *P* = 0.008), as well as between relative TW and %MPO improvement (*r* = 0.833, *P* < 0.001) (Figures 4A,E). No other significant correlations were observed: SpO_2_ and TRIMP (*r* = 0.281, *P* = 0.097; Figure 4B), TRIMP and relative TW (*r* = 0.118, *P* = 0.494; Figure 4C), SpO_2_ in hypoxic condition and %MPO improvement (*r* = 0.257, *P* = 0.336; Figure 4D), or TRIMP and %MPO improvement (*r* = 0.118, *P* = 0.494; Figure 4F).

## 4 Discussion

Six RSH sessions over two weeks produced significant training effects (Figures 1, 3A). However, inter-individual variability analysis revealed that performance improvement differed individually, with both responders and non-responders observed despite uniform RSH implementation (Supplementary Figure S1). Analysis of training parameters revealed a significant relationship between arterial deoxygenation and relative TW (Figure 4A), and a strong correlation between relative TW and %MPO improvement (Figure 4E). These findings suggest that differences in TW changes during training may lead to heterogeneous training outcomes, with varying effectiveness among participants.

### 4.1 Training outcomes

In this study, six sessions of RSH training (two sets of 5 × 10-s all-out cycle sprints; recovery: inter-sprint = 30 s, inter-set = 5 min; FiO_2_: 0.15) over two weeks resulted in a ∼3.8% increase in MPO and a ∼6.0% improvement in S_dec_ during repeated sprint tests (10 × 10-s all-out cycle sprints with passive 30-s recovery) in normoxia (Figures 1B, 3A). These performance benefits are consistent with previous RSH studies, which reported a ∼3% to 10% increase in MPO with similar training protocols (1–3 sets of 5–8 × 6–10 s sprints with 20–30 s recovery) and testing conditions (10 × 6–10 s sprints with 20–30 s recovery) under similar hypoxic conditions (FiO_2_: 0.141–0.162) (6, 12, 27). The slightly smaller improvements observed here might be attributed to the high training status of our sprint runners compared to less trained cohorts in previous studies, or the smaller training load (2 sets vs. 3–4 sets) used in this study (4). Although our observational study lacked a control group, our findings indicate that two sets of five all-out sprints (total 10 sprints per training session) might be sufficient for meaningful performance improvements. Despite the overall improvement, there was considerable inter-individual variability, with ∼20% of participants showing no performance improvement (i.e., non-responders) after the same RSH training (Supplementary Figure S1).

During pre- and post-test assessments, heart rate significantly increased across repetitions, with no effect of training (Figure 2). This result aligns with previous studies showing no cardiovascular adaptations from RSH (6, 28–30). Peripheral skeletal muscle adaptations (e.g., increased capillary-to-fiber ratio, myoglobin content, and oxidative enzyme activity) are likely the main adaptations to repeated maximal-intensity hypoxic training (15, 16). Previous studies have also indicated that hypoxic training can increase glycolytic enzyme activity, especially phosphofructokinase (31, 32). However, this study did not show an increase in blood lactate concentrations after the training intervention (Figure 3B), aligning with observations from previous RSH studies (6, 12, 29, 30). During repeated sprints, the glycolytic system is highly active initially, but its contribution decreases with successive efforts, leading to a plateau in blood lactate levels (3, 33). Hence, potential differences in glycolytic enzyme activity may not be reflected in blood lactate concentrations due to this plateau effect.

### 4.2 Inter-individual variability

We observed inter-individual variability in performance improvements after the RSH intervention, including the presence of non-responders (Supplementary Figure S1). Although the effectiveness of RSH has been well demonstrated in meta-analyses (4), the presence of inter-individual variability in performance benefits and non-responders indicates that the present RSH protocol may not benefit all athletes equally. Gutknecht et al. (12) reported that SpO₂ fluctuations during RSH training sessions were significantly correlated with performance improvements under varying hypoxic exposures (FiO₂: 0.141, 0.162, or 0.175). In contrast, our study demonstrates that this phenomenon also occurs under identical hypoxic conditions (FiO_2_: 0.150), indicating that it may be driven by inter-individual responses to hypoxia. To explore the cause of these heterogenous training effects, we examined the relationship between individual performance improvement (%MPO improvement) and training parameters (SpO_2_, TRIMP and relative TW; Figure 4). Internal hypoxic response in blood level (SpO_2_) during training sessions significantly correlated with relative TW (Figure 4A), indicating that participants with greater arterial deoxygenation achieved lower TW during hypoxic training sessions. Consistent with these observations, our previous research showed significant individual variations in SpO_2_ levels (ranging from 91.6% to 82.2%), with those experiencing greater declines in SpO_2_ also showing larger reductions in RSA under moderate hypoxia (FiO_2_: 0.15) (11). Increasing severity of arterial hypoxemia resulted in greater peripheral muscle fatigue during repeated sprint exercise in severe hypoxia (FiO_2_: 0.13) compared to normoxia (FiO_2_: 0.21) and moderate hypoxia (FiO_2_: 0.17) (34). Thus, decreased mechanical output during training (relative TW) in individuals with greater arterial deoxygenation may primarily be attributed to peripheral fatigue. Peripheral muscle fatigue induced by arterial hypoxemia may be associated with a decline in tissue oxygenation; however, this remains a topic for future investigation.

There was a significant correlation between relative TW and %MPO improvement (Figure 4E), indicating that participants with lower relative TW (mechanical output) derived fewer benefits from RSH. This aligns with previous studies linking reduced mechanical output to diminished activation of the AMPK pathway and smaller training gains (18–20). Taken together, excessive arterial deoxygenation during training sessions may decrease relative TW (mechanical output), potentially compromising performance improvement. This is supported by research on RSH under various hypoxic conditions (FiO_2_: 0.141, 0.162 or 0.175), which reported a significant correlation between SpO_2_ during training and performance improvement (12). Specifically, greater decreases in SpO_2_ were associated with smaller performance gains (12). Interestingly, performance improvements occurred only under mild hypoxia (FiO_2_: 0.162–0.175), where arterial deoxygenation was mild (SpO_2_: ∼93%–89%), but not under severe hypoxia (FiO2: 0.141) with large arterial deoxygenation (SpO_2_: ∼82%), indicating that excessive arterial deoxygenation may negatively affect performance outcomes (12). However, in our study, there was no significant correlation between SpO_2_ and %MPO improvement (Figure 4D), indicating that SpO_2_ variability is not the sole determinant of performance, and other factors may also influence training outcomes. One possible explanation is that a decrease in SpO₂ may reduce mechanical stress while increasing physiological stress, potentially promoting adaptations through mechanisms such as HIF activation (13, 14).

There was no significant correlation between SpO_2_ and TRIMP (Figure 4B), indicating that a decrease in SpO_2_ did not affect cardiovascular strain during RSH. This aligns with previous studies, which observed no significant differences in heart rate responses between hypoxia and normoxia, suggesting that changes in arterial deoxygenation did not alter cardiovascular solicitation during RSH (28, 35). TRIMP also did not correlate with %MPO improvement (Figure 4F), further suggesting that differences in cardiovascular responses had minimal impact on RSH training effectiveness. In contrast, previous research has shown that heart rate can decrease with increasing hypoxia due to compensatory vasodilation (36). Although moderate hypoxia (FiO_2_: 0.15) was applied in this study, heart rate responses may differ with more severe hypoxia.

### 4.3 Limitations

We recruited highly-trained athletes who maintained their regular training outside of the prescribed RSH intervention. While this incidental training (not quantified) may have influenced the results, all participants were from the same team and followed a similar training regimen during the same pre-competition phase. Several studies have consistently demonstrated positive effects when elite athletes combine RSH with their “normal” training routines (15, 16, 27, 28). Therefore, examining the combination of RSH and regular training is crucial for understanding its practical applications in real-world settings.

This study is an observational experiment involving highly trained athletes, and the absence of a comparison group (e.g., a normoxic training group) represents a significant limitation. Due to ethical and logistical considerations, including a normoxic training group that would not receive the potential benefits of the RSH intervention was not feasible for highly trained athletes. To examine inter-individual variability in response to the RSH intervention, we conducted a test-retest assessment to estimate potential random noise from the intervention. While this approach helped mitigate the impact of lacking a comparison group, future research should include a randomized controlled trial with a comparison group and female participants to provide more robust evidence.

## 5 Conclusion

Six RSH sessions performed over two weeks led to significant performance improvements during repeated sprint tests, with a ∼3.8% increase in MPO and a ∼6.0% reduction in S_dec_. Inter-individual variability analysis revealed that performance improvements differed between individuals, with nearly 20% of participants not experiencing any performance benefit from the intervention. The internal hypoxic response (SpO_2_) showed a significant correlation with relative TW, which was strongly associated with performance improvement. Taken together, training parameters such as arterial deoxygenation and relative TW in highly-trained sprint runners may contribute to the observed heterogeneous training effects.

## Data Availability

The raw data supporting the conclusions of this article will be made available by the authors, without undue reservation.

## References

[1] Girard O Brocherie F Goods PSR Millet GP. An updated panorama of “living low-training high” altitude/hypoxic methods. Front Sports Act Living. (2020) 2:26. 10.3389/fspor.2020.00026 33345020 PMC7739748

[2] Wilber RL. Application of altitude/hypoxic training by elite athletes. Med Sci Sports Exerc. (2007) 39(9):1610–24. 10.1249/mss.0b013e3180de49e6 17805095

[3] Girard O Mendez-Villanueva A Bishop D. Repeated-sprint ability—part I: factors contributing to fatigue. Sports Med. (2011) 41(8):673–94. 10.2165/11590550-000000000-00000 21780851

[4] Brocherie F Girard O Faiss R Millet GP. Effects of repeated-sprint training in hypoxia on sea-level performance: a meta-analysis. Sports Med. (2017a) 47(8):1651–60. 10.1007/s40279-017-0685-3 28194720

[5] Faiss R Girard O Millet GP. Advancing hypoxic training in team sports: from intermittent hypoxic training to repeated sprint training in hypoxia. Br J Sports Med. (2013a) 47 Suppl 1(Suppl 1):i45–50. 10.1136/bjsports-2013-092741 24282207 PMC3903143

[6] Faiss R Léger B Vesin J-M Fournier P-E Eggel Y Dériaz O Significant molecular and systemic adaptations after repeated sprint training in hypoxia. PLoS One. (2013b) 8(2):e56522. 10.1371/journal.pone.0056522 23437154 PMC3577885

[7] Soo J Girard O Ihsan M Fairchild T. The use of the SpO_2_ to FiO_2_ ratio to individualize the hypoxic dose in sport science, exercise, and health settings. Front Physiol. (2020) 11:570472. 10.3389/fphys.2020.570472 33329021 PMC7714921

[8] Albert TJ Swenson ER. Peripheral chemoreceptor responsiveness and hypoxic pulmonary vasoconstriction in humans. High Alt Med Biol. (2014) 15(1):15–20. 10.1089/ham.2013.1072 24444139

[9] Chapman RF. The individual response to training and competition at altitude. Br J Sports Med. (2013) 47 Suppl 1(Suppl 1):i40–4. 10.1136/bjsports-2013-092837 24282206 PMC3903142

[10] Chapman RF Stager JM Tanner DA Stray-Gundersen J Levine BD. Impairment of 3000-m run time at altitude is influenced by arterial oxyhemoglobin saturation. Med Sci Sports Exerc. (2011) 43(9):1649–56. 10.1249/MSS.0b013e318211bf45 21311361

[11] Takei N Muraki R Girard O Hatta H. Inter-individual variability in peripheral oxygen saturation and repeated sprint performance in hypoxia: an observational study of highly-trained subjects. Front Sports Act Living. (2024) 6:1452541. 10.3389/fspor.2024.1452541 39176235 PMC11338753

[12] Gutknecht AP Gonzalez-Figueres M Brioche T Maurelli O Perrey S Favier FB. Maximizing anaerobic performance with repeated-sprint training in hypoxia: in search of an optimal altitude based on pulse oxygen saturation monitoring. Front Physiol. (2022) 13:1010086. 10.3389/fphys.2022.1010086 36311239 PMC9597871

[13] Goods PSR Billaut F Brocherie F Louis J. Editorial: managing physiological and biomechanical load-adaptation pathways in high performance sport: challenges and opportunities. Front Sports Act Living. (2022) 4:1041998. 10.3389/fspor.2022.1041998 36263293 PMC9574354

[14] Semenza GL. Hypoxia-inducible factor 1: master regulator of O2 homeostasis. Curr Opin Genet Dev. (1998) 8(5):588–94. 10.1016/S0959-437X(98)80016-6 9794818

[15] Brocherie F Millet GP D’Hulst G Van Thienen R Deldicque L Girard O. Repeated maximal-intensity hypoxic exercise superimposed to hypoxic residence boosts skeletal muscle transcriptional responses in elite team-sport athletes. Acta Physiol. (2018) 222(1):e12851. 10.1111/apha.12851 28103427

[16] Pramkratok W Songsupap T Yimlamai T. Repeated sprint training under hypoxia improves aerobic performance and repeated sprint ability by enhancing muscle deoxygenation and markers of angiogenesis in rugby sevens. Eur J Appl Physiol. (2022) 122(3):611–22. 10.1007/s00421-021-04861-8 34977961

[17] Goods P SR Dawson BT Landers GJ Gore CJ Peeling P. Effect of different simulated altitudes on repeat-sprint performance in team-sport athletes. Int J Sports Physiol Perform. (2014) 9(5):857–62. 10.1123/ijspp.2013-0423 24509626

[18] Hickson RC Foster C Pollock ML Galassi TM Rich S. Reduced training intensities and loss of aerobic power, endurance, and cardiac growth. J Appl Physiol. (1985) 58(2):492–9. 10.1152/jappl.1985.58.2.492 3156841

[19] Levine BD Stray-Gundersen J. “Living high-training low”: effect of moderate-altitude acclimatization with low-altitude training on performance. J Appl Physiol. (1997) 83(1):102–12. 10.1152/jappl.1997.83.1.102 9216951

[20] Wadley GD Lee-Young RS Canny BJ Wasuntarawat C Chen ZP Hargreaves M Effect of exercise intensity and hypoxia on skeletal muscle AMPK signaling and substrate metabolism in humans. Am J Physiol Endocrinol Metab. (2006) 290(4):E694–702. 10.1152/ajpendo.00464.2005 16263768

[21] Atkinson G Batterham AM. True and false interindividual differences in the physiological response to an intervention. Exp Physiol. (2015) 100(6):577–88. 10.1113/EP085070 25823596

[22] Bonafiglia JT Preobrazenski N Gurd BJ. A systematic review examining the approaches used to estimate interindividual differences in trainability and classify individual responses to exercise training. Front Physiol. (2021) 12:665044. 10.3389/fphys.2021.665044 34819869 PMC8606564

[23] Swinton PA Hemingway BS Saunders B Gualano B Dolan E. A statistical framework to interpret individual response to intervention: paving the way for personalized nutrition and exercise prescription. Front Nutr. (2018) 5:41. 10.3389/fnut.2018.00041 29892599 PMC5985399

[24] McKay AKA Stellingwerff T Smith ES Martin DT Mujika I Goosey-Tolfrey VL Defining training and performance caliber: a participant classification framework. Int J Sports Physiol Perform. (2022) 17(2):317–31. 10.1123/ijspp.2021-0451 34965513

[25] Banister EW. Modeling elite athletic performance. In: MacDougall JD Wenger HA Green HJ, editors. Physiological Testing of Elite Athletes. Champaign. Illinois: Human Kinetics (1991). p. 403–24.

[26] Atkinson G Nevill AM. Statistical methods for assessing measurement error (reliability) in variables relevant to sports medicine. Sports Medicine (Auckland. N.Z*)*. (1998) 26(4):217–38. 10.2165/00007256-199826040-00002 9820922

[27] Kasai N Mizuno S Ishimoto S Sakamoto E Maruta M Kurihara T Impact of six consecutive days of sprint training in hypoxia on performance in competitive sprint runners. J Strength Cond Res. (2019) 33(1):36–43. 10.1519/JSC.0000000000001954 28445224

[28] Brocherie F Millet GP Girard O. Psychophysiological responses to repeated-sprint training in normobaric hypoxia and normoxia. Int J Sports Physiol Perform. (2017) 12(1):115–23. 10.1123/ijspp.2016-0052 27139930

[29] Galvin HM Cooke K Sumners DP Mileva KN Bowtell JL. Repeated sprint training in normobaric hypoxia. Br J Sports Med. (2013) 47 Suppl 1(Suppl 1):i74–9. 10.1136/bjsports-2013-092826 24282212 PMC3903144

[30] Shi Q Tong TK Nie J Tao D Zhang H Tan X Repeated-sprint training in hypoxia boosts up team-sport-specific repeated-sprint ability: 2-week vs 5-week training regimen. Eur J Appl Physiol. (2023) 123(12):2699–710. 10.1007/s00421-023-05252-x 37335354

[31] Hoppeler H Vogt M. Muscle tissue adaptations to hypoxia. J Exp Biol. (2001) 204(Pt 18):3133–9. 10.1242/jeb.204.18.3133 11581327

[32] Puype J Van Proeyen K Raymackers J-M Deldicque L Hespel P. Sprint interval training in hypoxia stimulates glycolytic enzyme activity. Med Sci Sports Exerc. (2013) 45(11):2166–74. 10.1249/MSS.0b013e31829734ae 23604068

[33] Parolin ML Chesley A Matsos MP Spriet LL Jones NL Heigenhauser GJ. Regulation of skeletal muscle glycogen phosphorylase and PDH during maximal intermittent exercise. Am J Physiol. (1999) 277(5):E890–900. 10.1152/ajpendo.1999.277.5.E890 10567017

[34] Townsend N Brocherie F Millet GP Girard O. Central and peripheral muscle fatigue following repeated-sprint running in moderate and severe hypoxia. Exp Physiol. (2021) 106(1):126–38. 10.1113/EP088485 32557892

[35] Gatterer H Menz V Untersteiner C Klarod K Burtscher M. Physiological factors associated with declining repeated sprint performance in hypoxia. J Strength Cond Res. (2019) 33(1):211–6. 10.1519/JSC.0000000000001891 28277432

[36] Casey DP Joyner MJ. Compensatory vasodilatation during hypoxic exercise: mechanisms responsible for matching oxygen supply to demand. J Physiol (Lond). (2012) 590(24):6321–6. 10.1113/jphysiol.2012.242396 22988134 PMC3533194

