# Supplementary material for: Inter-individual variability in performance benefits from repeated sprint training in hypoxia and associated training parameters
Source: Front Sports Act Living. 2025 Apr 15;7:1524437. doi: 10.3389/fspor.2025.1524437 (PMC12037587; doi:10.3389/fspor.2025.1524437)
Supplement: Supplementary file 1 [file Datasheet1.pdf]

Supplementary Material

1 Supplementary Figures and Tables

1.1 Supplementary Figure S1

Individual pre-post performance improvement

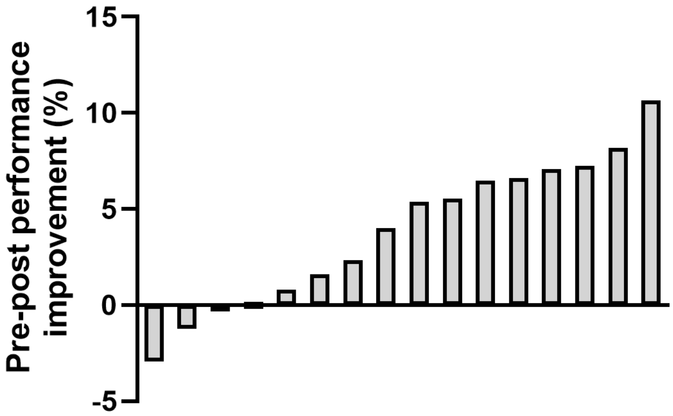

Each bar indicates individual values of pre-post performance improvement.

1.2 Supplementary Table S1.

Characteristics of participants

	Age (Year)	Weight (kg)	Height (m)	Personal best records (s)		
				100-m (n = 10)	200-m (n = 6)	400-m (n = 8)
Participants (n = 16)	20.2 ± 1.7	66.9 ± 7.2	1.74 ± 0.07	10.81 ± 0.26	21.55 ± 0.61	49.27 ± 1.56
Range	19-25	55.4-79.2	1.63-1.86	10.33-11.20	20.86-22.35	45.81-50.79

Values are means ± SD.
